# Editorial: Global excellence in cellular immunology: Europe 2021

**DOI:** 10.3389/fimmu.2023.1283105

**Published:** 2023-09-07

**Authors:** Markus Uhrberg, Andreas Radbruch

**Affiliations:** ^1^ Institute of Transplantation Diagnostics and Cell Therapeutics, University Hospital of Düsseldorf, Düsseldorf, Germany; ^2^ Deutsches Rheuma-Forschungszentrum Berlin, a Leibniz Institute, Berlin, Germany

**Keywords:** T cells, NK cells, tissue residency, memory, checkpoint inhibition

This Research Topic was set up to highlight recent progress in cellular immunology in Europe. Rather than focus on a particular smaller topic, it aims to shed light on recent progress made across the entire breadth of the cellular immunology field in Europe. Considering this, the present Research Topic hosts a small but nice collection of four articles highlighting the present key aspects of T cell and Natural Killer (NK) cell immunology: memory compartments, tissue-residency, checkpoint regulation, and mechanism of cytotoxicity. Of note, all articles focus on the human immune system, which we in general do understand less well than the murine immune system. Given the genetic and functional differences between human and mouse immune cells already known (1, 2), the need for a better understanding of the human immune system is obvious, in order to solve the many burning questions involved in avoiding and controlling infectious diseases, autoimmunity, and cancer. The field is hampered by the logistic and ethical constraints that are attached to working with tissue biopsies other than blood from patients or even harder, from healthy individuals. The research work presented here reflects the ongoing efforts to better understand how the human immune system works on the systemic level.

In the study by Haugstøyl et al., the surface proteome of NK cells residing in adipose tissue was characterized. Adipose tissue-resident NK cells have recently gained much interest from studies suggesting them to be mediators of inflammation and insulin resistance in obesity (3). So far, NK cells residing in human adipose tissue are poorly defined. The present study performed high-dimensional flow cytometric analyses on NK cells from subcutaneous (SAT) as well as visceral adipose tissue (VAT). The authors nicely illustrate how tissue-resident NK cells adapt to their specific microenvironment by defining protein signatures specific for VAT vs. SAT. Notably, VAT contained a higher frequency of immature tissue-resident NK cells compared to SAT, which is interesting given that the VAT/SAT ratio correlates with risk of cardiovascular disease and insulin resistance (4). Although a link between the presence of NK cell subsets and insulin resistance has not yet been established in the present study, the newly defined surface markers will be a valuable asset to explore if and how NK cells contribute to obesity-induced inflammatory stress and diabetes.

Another type of lymphocyte, absent from peripheral blood and a challenge to analyze, are human tissue-resident memory T cells (T_rm_) (5). When T cells enter a nonlymphoid organ in the effector phase of an immune response, a subset of them can develop *in situ* into T_rm_ cells that remain in the tissue and do not recirculate anymore. T_rm_ cells ensure their tissue-residency by acquisition of a specific set of receptors that varies between organs but typically involves upregulation of CD69 and downregulation of S1PR1 as well as other receptors enabling recirculation such as CCR7. In the tissue, upon reinfection, T_rm_ can mount rapid antigen-specific recall responses, i.e. immunosurveillance at an early stage of infection. The focus of the present review by Ginsberg et al., are T_rm_ cells residing in the kidney. The work illustrates the Janus-faced nature of this highly-sensitive memory T cell compartment, which provides antigen-specific immune protection against pathogens, but if autoreactive, can also promote autoimmune diseases. The review summarizes our knowledge on the role of pathogenic renal T_rm_ in glomerulonephritis, emphasizing that most T cells in the kidney have a T_rm_ phenotype and that their abundance correlates with disease severity. Finally, the authors discuss possible strategies to target pathogenic T_rm_ in renal autoimmune disease based on metabolic inhibition, cytokine deprivation, or induction of antigen-specific tolerance with autoantigen-coated reagents.

Besides tissue resident T_rm_, effector memory (T_em_) and central memory (T_cm_) T cells circulating through blood and lymph provide systemic protection, by patrolling secondary lymphoid organs and migrating to inflamed nonlymphoid tissues (6). It is believed that T_em_ are more cytotoxic than T_cm_ cells, but a quantitative comparison including dissection of the cytotoxic mechanisms on the single cell level has been lacking. In their present work, Knörck et al. describe a FRET (Förster Resonance Energy Transfer)-based assay to distinguish between target cell apoptosis and necrosis on the single cell level. The study exemplifies the importance of cooperation across system boundaries, in this case a team of biophysicists employing a FRET reporter system developed by chemists (7) and applying it to basic immunology questions. The authors used a Casper3 FRET reporter with a caspase 3 cleavage site to monitor apoptosis and necrosis event, simultaneously tracking effector/target cell contact times by real-time microscopy. T_em_ were more efficient killers than T_cm_ and this correlated to higher perforin content, faster target cell contact and more efficient immune synapse formation. The new technique will aid to evaluate how different T cell subsets, for example in CAR-T cell therapy, contribute to tumor control.

Umbilical cord blood (CB) is another home of immune and hematopoietic cells of basic and applied interest. It not only is a source for allogeneic stem cells used for transplantation, but also holds great promise as a source for cell-based tumor therapy and the *in vitro* generation of immune cells with therapeutic potential that are rare in peripheral blood such as innate lymphoid cells (ILCs) and plasmacytoid dendritic cells (8–11). Here, Greppi et al. have identified a novel subpopulation of NK cells in CB that express the checkpoint inhibitor programmed death receptor 1 (PD1). The same group had previously shown that so-called adaptive NK cells, a population of memory NK cells specifically arising upon infection with human Cytomegalovirus (HCMV) could express PD-1 (12). It thus came as a surprise that PD1^+^ NK cells are also present in CB, which except for rare cases of mother-to-child transmission, had never been exposed to HCMV. Importantly, the authors were able to obtain CB from the child together with peripheral blood from the mother and could show that PD1^+^ NK cells arise independent of HCMV seropositivity of the mother. Notably, PD1^+^ NK cells were generally more responsive to target cell and cytokine stimulation whereas direct triggering of PD1 led to inhibition of PD1^+^ NK cells as expected. Given that PD1 checkpoint inhibition is widely used in treatment of cancer, it will be important to find out in how far this affects tumor-specific responses of CB-derived NK cells.

Altogether, the different contributions in this Research Topic provide an excellent display of recent advances in cellular immunology in Europe.

## Author contributions

MU: Conceptualization, Writing – original draft, Writing – review & editing. AR: Conceptualization, Writing – original draft, Writing – review & editing.
